# ApoE-fragment/Aβ heteromers in the brain of patients with Alzheimer’s disease

**DOI:** 10.1038/s41598-019-40438-4

**Published:** 2019-03-08

**Authors:** Amandine Mouchard, Marie-Charlotte Boutonnet, Claire Mazzocco, Nathalie Biendon, Nathalie Macrez, Neuro-CEB Neuropathology Network

**Affiliations:** 1 Bordeaux University, Institut des Maladies Neurodégénératives, UMR, 5293 Bordeaux, France; 2 CNRS, Institut des Maladies Neurodégénératives, UMR, 5293 Bordeaux, France

## Abstract

Identification of endogenous pathological amyloid β peptides (Aβ) forms in the brains of patients with Alzheimer’s disease (AD) is still unclear. In healthy brain, Aβ can associate with Apolipoprotein E (ApoE) which is involved in its metabolism and clearance. In the brain of patients with AD, ApoE is cleaved and produces ApoE fragments. We studied the forms of Aβ and their interaction with the ApoE fragments in post-mortem brains from control and AD patients by western blots and co-immunoprecipitation. Three Aβ-containing peptides and three ApoE fragments were specifically found in the brain of AD patients. Co-immunoprecipitations showed that ApoE fragments and Aβ1–42 peptides are co-partners in heteromers of 18 and 16 kDa while ApoE-fragments and Aβ peptides of 12 kDa did not interact with each other. Formation of the 18 kDa ApoE-fragment/Aβ heteromers is specifically increased in ApoE4 carriers and is a strong brain marker of AD while 16 kDa ApoE-fragment/Aβ and Aβ 12 kDa correlate to memory deficit. These data show that in patients with AD, ApoE fragmentation generates peptides that trap Aβ in the brain. Inhibiting the fragmentation or targeting ApoE fragments could be exploited to define strategies to detect or reverse AD.

## Introduction

Research on Alzheimer disease (AD) has mainly focused on the role of β-amyloid peptide (Aβ) and on the imbalance between production and clearance of Aβ^1^. Over 40 soluble Aβ peptides have been found in cell culture medium^2^ and Aβ is biologically present in every human brain, its concentration being increased in people with AD^3,4^. Beside Aβ1-42, there are many types of Aβ peptides, including N-terminal-extended peptides and amino- or carboxy-truncated peptides^1,5–7^. In addition, Aβ can exhibit different aggregation states including as monomer, dimer, oligomer, fibril or as heteromer when it associates with other proteins. Therefore, an understanding of the different forms of Aβ across the different sequence lengths, aggregation states and neuropathological associations is still required^1,8^. Many scientists studying AD-related Aβ oligomers work with mouse models of AD or with *in vitro* synthesized Aβ oligomers and found or made different types of oligomers^5,9^. The few studies that have analyzed post-mortem human brain samples from AD-labeled patients resulted in the identification of dimers, trimers^10^, dodecamers^11,12^ or tetra/pentamers^13,14^ that appear or are increased in AD patients compared to controls. These studies use different extractions, different antibodies and samples whose classification as AD was based on different criteria: (i) high Braak stage^12^; (ii) high Braak stage and deficient cognitive status^13,14^; (iii) total post-mortem Aβ42 measured in the brains of patients by ELISA^10^. Here, we selected patients with cognitive impairment and high levels of both Aβ plaques and Tau tangles as representative cases of AD and studied Aβ and Apolipoprotein E (ApoE) expression in their brain.

Indeed, ApoE influences the brain transport and elimination of lipids and Aβ^15,16^ and is thought to play many roles in AD^17,18^. ApoE binds to Aβ and regulates its metabolism, clearance, aggregation, and deposition^19–22^. Among the three human ApoE isoforms, inheritance of the *APOE ε4* (*APOE4*) allele is the strongest known risk factor for AD besides age^23,24^. In human ApoE4-expressing familial AD-transgenic mouse model, Aβ-ApoE interactions are lower and oligomeric Aβ levels are higher than in other human ApoE-expressing mice^25^. Targeting of nonlipidated, aggregated ApoE with antibodies inhibits amyloid accumulation in a transgenic mice model of AD^26^ suggesting that targeting ApoE could be exploited to prevent or reverse the Aβ pathology of AD^27^.

ApoE fragmentation also arose as a potential AD-related pathological process. Indeed, carboxy-terminal-truncated forms of ApoE, found in AD brains and in cultured neurons, induce intracellular neurofibrillary tangle-like inclusions in neurons^28–30^. Carboxy-terminal ApoE peptides stabilize the formation of Aβ oligomers *in vitro*^31^ and amino-terminal ApoE peptides help binding of Aβ to α7-nACh receptor^32^. However, the relation between ApoE fragments and Aβ forms has not been studied so far in human brain.

Here, we studied native forms of Aβ and ApoE in post-mortem human brain tissues of AD or control patients. We used non-denaturant protein extraction and western blot analyses to characterize the peptides with a panel of antibodies targeting various sites on Aβ or ApoE. We also analyzed the Aβ-ApoE interactions in the brains of patients with AD according to their *APOE* genotype.

## Results

### Aβ forms in the brain of patients with AD

**Figure 1:**
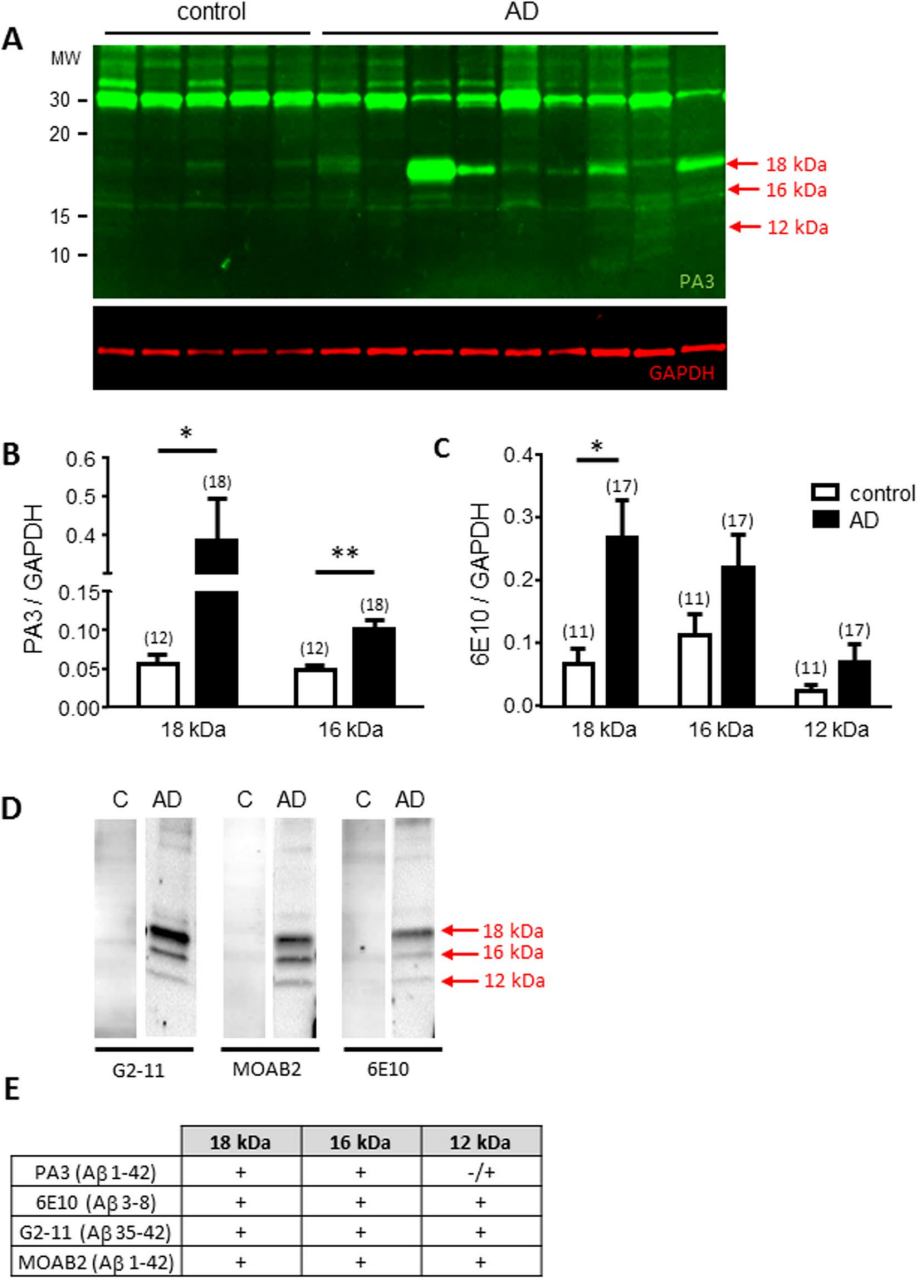
Quantification and characterization of the Aβ forms found in the cortex of AD patients. (**A)** Representative western blot of cortex proteins extracted from control and AD patients (each pit representing a different patient) and revealed with an anti-Aβ full length (PA3-16761, green). GAPDH (red) was revealed as an internal control for each deposit. (**B**) Bar graph representing the statistical analysis of Aβ/GAPDH ratio measured with PA3 antibody for 18 and 16 kDa forms in control *versus* AD brain samples. (**C**) Bar graphs representing the statistical analysis of the Aβ/GAPDH ratio measured with 6E10 antibody for the 18, 16 and 12 kDa forms in control *versus* AD brain samples. The number of samples analyzed is indicated in parentheses, significant statistical differences are marked by *(p < 0.05) and **(p < 0.005) as calculated by Mann-Whitney test. (**D**) Western blots of cortex from a control (Braak 1, Thal 0) and an AD patient (Braak 6, Thal 4) revealed with 3 different anti-Aβ antibodies (G2-11, MOAB2, 6E10). All fours antibodies recognized the 18 and 16 kDa forms of Aβ as compiled in (**E**) while the PA3 antibody poorly recognize the 12 kDa Aβ form. The lanes illustrated in Fig. 1D have been cropped from Supplementary Fig. S3A.

Three forms of Aβ 18, 16 and 12 kDa were specifically found in the cortex of AD *versus* control patients (Fig. 1A,D). The amount of each Aβ form was quantified by using different anti-Aβ antibodies and statistical analyses show that the 18 kDa Aβ peptides significantly increase in AD *versus* control brain (Fig. 1B; p = 0.0059 with PA3 and Fig. 1C; p = 0.0042 with 6E10), the 16 kDa Aβ peptides increase in AD *versus* control brain when measured with PA3 (Fig. 1B; p = 0.0007) but is not significant when measured with 6E10 (Fig. 1C; p = 0.2337). The lower molecular weight 12 kDa form is found with 6E10 antibody (Fig. 1C,D) antibody but not with PA3 (Fig. 1A,B). Statistical analysis of 12 kDa peptides show that the increase observed in AD patient is not statistically different from control patients (Fig. 1C; p = 0.1355). The majority 18 kDa Aβ form was also increased in hippocampus of AD patients (Supplementary Fig. S2).

Characterization of the 18, 16 and 12 kDa Aβ forms found in the cortex of AD patients was achieved by using 2 additional anti-Aβ antibodies which help to determine the contours of the identified Aβ peptides. N-terminus of Aβ1-42 was revealed by 6E10 positivity while G2-11 positivity revealed the C-terminus of Aβ1-42; PA3 and MOAB2 both recognized the full length human Aβ1-42. All four antibodies recognized the 18 and 16 kDa forms of Aβ (Fig. 1D,E) showing that full length Aβ1-42 is present in 16 and 18 kDa Aβ peptides. Although consisting of Aβ1-42 peptide as well, Aβ 12 kDa probably has a macromolecular structure that makes it poorly recognizable by PA3 (Fig. 1A,D,E).

Aβ1-42 monomers were not detected in the brain of patients with AD by any of the antibodies we used. In contrast, the same antibodies identified monomeric Aβ 4 kDa when purified Aβ1-42 was used as external standard or in the brain of APPxPS1 mice (Supplementary Fig. S1A). The 6E10 and PA3 antibodies also revealed Aβ 4/8 kDa in human cortex of AD patient that were either degraded by leaving the proteins overnight at room temperature or prepared in denaturant conditions with SDS 2% (Supplementary Fig. S1B,C). These control experiments show that the 4 kDa Aβ form can be observed only when human brain samples were modified by experimental conditions or in brain samples of APPxPS1 mice which overproduce Aβ.

### Correlation of the Aβ forms with MMSE

**Figure 2:**
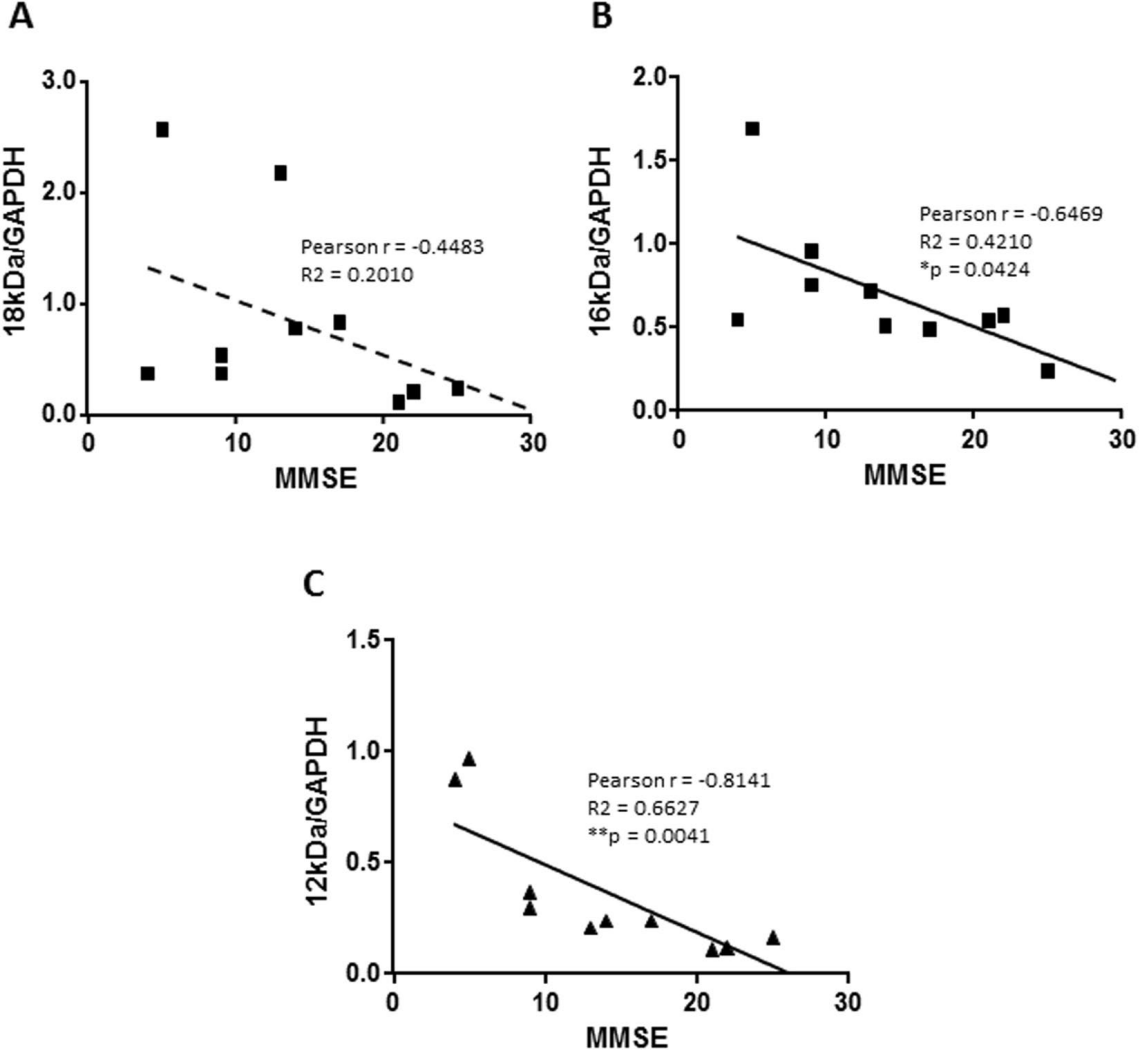
Correlation between Aβ forms and the cognitive status of the patients evaluated by MMSE. Quantitative analyses of Aβ forms (6E10 relative to the quantity of GAPDH for each sample) were plotted in function of the MMSE score for each patient. The statistical analysis of correlation between the quantity of 18, 16 and 12 kDa Aβ forms and MMSE as well as the linear regressions of the data are plotted in (**A**–**C**) respectively. Pearson analyses reveal that the quantities of the 16 kDa and 12 kDa Aβ forms are significantly correlated to the cognitive status of the patients while the amount of the 18 kDa Aβ form is not correlated to the MMSE. Significant correlation are marked by *(p < 0.05) and **(p < 0.005).

We evaluated the correlation between Aβ forms and the cognitive status of ten patients. Quantitative analyses of Aβ forms (6E10/GAPDH) were plotted in function of the MMSE score for each patient (Fig. 2). The statistical analysis of Pearson correlation between the quantity of 18, 16 or 12 kDa Aβ forms and MMSE score revealed that the quantities of the 16 and 12 kDa Aβ forms are significantly correlated to the cognitive status of the patients (p = 0.0424 and p = 0.0041, respectively) while the amount of the 18 kDa Aβ form is not correlated to the MMSE. These results suggest that the main 18 kDa form could be a storage or preservative form of Aβ while the 16 and 12 kDa Aβ forms could be toxic for the brain and impair memory. The correlation graphs (Fig. 2B,C) show that Aβ 16 and 12 kDa correlate better with middle MMSE (10 < MMSE < 25) than with very low MMSE (<10).

### ApoE forms in the brains of patients with AD

**Figure 3:**
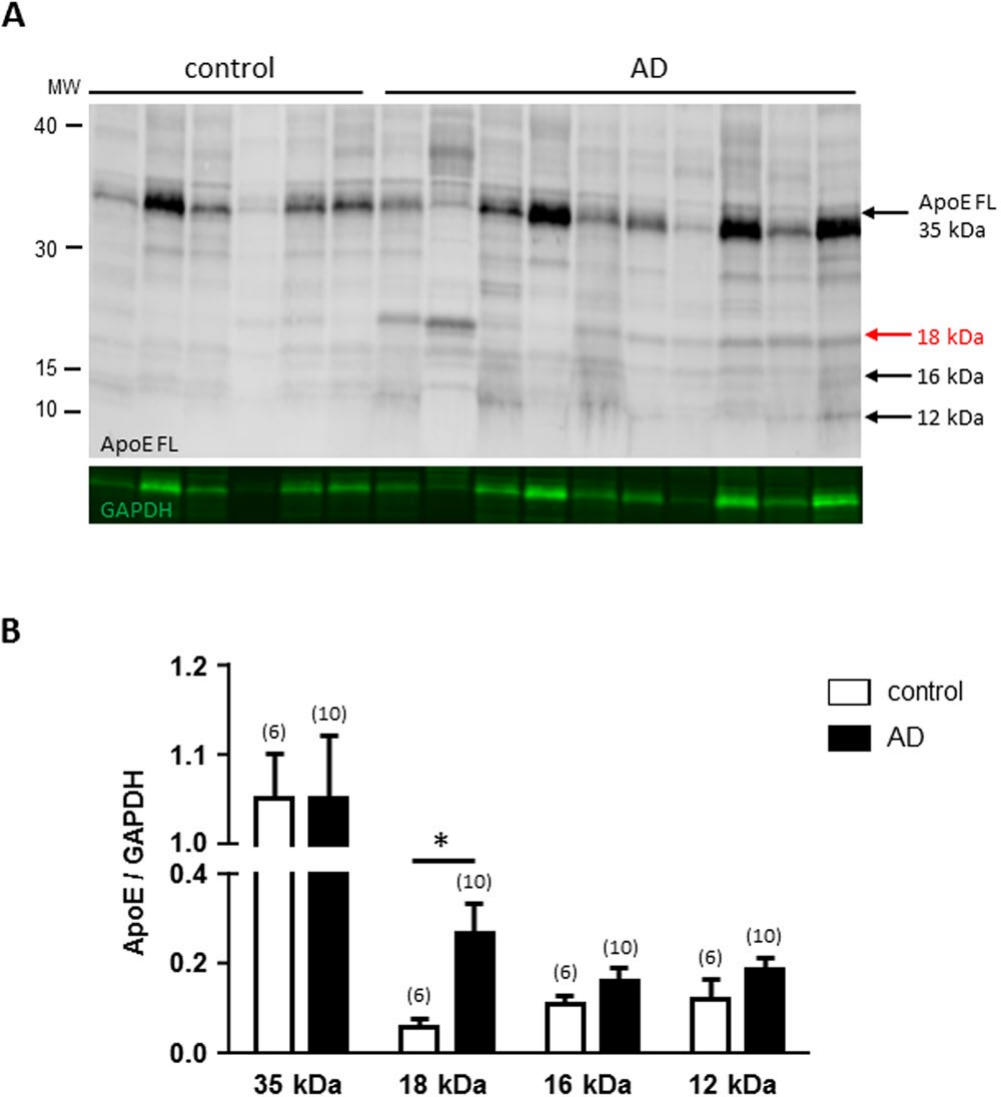
ApoE protein and fragments detected in the cortex of AD patients. **(A)** Representative western blot of cortex proteins extracted from control and AD patients and revealed with an anti-ApoE full length antibody (178479 Calbiochem). The full length ApoE protein (35 kDa) was found in all samples and 3 additional forms (18, 16 and 12 kDa) were found only in the cortex of AD patients. (**B**) Bar graphs representing the statistical analysis of the quantification of various ApoE forms for each sample. The number of samples analyzed is indicated in parentheses, significant statistical differences between control and AD are marked by *(p < 0.05) as calculated by Mann-Whitney Test.

Since ApoE can increase Aβ oligomer formation^20,21^ and truncated forms of ApoE occur in AD brains^30^, we investigated the multiple forms of ApoE present in the brain of control and AD patients. The full length ApoE protein (35 kDa) and a shorter ApoE protein of 30 kDa were found in all samples but three additional forms (18, 16 and 12 kDa) were specifically found in the cortex of AD patients as shown in Fig. 3A. Statistical analyses show that only the ApoE 18 kDa was significantly increased in AD patients (Fig. 3B, p = 0.0066) while the increases in 16 and 12 kDa ApoE were not significant (p = 0.3673 and p = 0.1179, respectively). As for Aβ, the forms of ApoE fragments were altered when the proteins were degraded after overnight at room temperature which results in the loss of the 18 and 16 kDa forms (Supplementary Fig. S1D). The ApoE 12 kDa form seems less sensitive to degradation.

**Figure 4:**
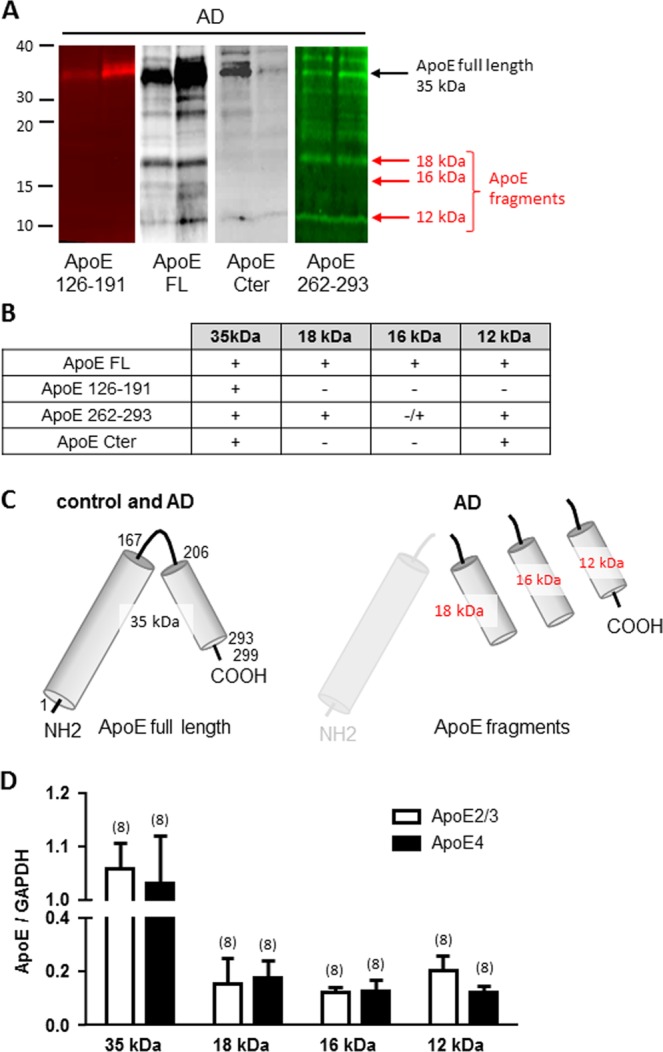
Characterization of ApoE fragments detected in the cortex of AD patients. **(A**) Illustrations of western blots of cortex from 2 AD patients revealed with 4 different anti-ApoE antibodies (ApoE 126-191, ApoE FL, ApoE Cter and ApoE 262–293). All fours antibodies recognized the 35 kDa form corresponding to the full length ApoE but only the anti-ApoE FL and the anti-ApoE 262–293 identified the 18 and 16 kDa forms, the smaller 12 kDa form was identified by three ApoE antibodies as compiled in (**B**,**C**). Schematic illustration of ApoE in control patient and of ApoE fragments formed only in AD patients. (**D**) Bar graph illustrating the effect of ApoE2/3 carriers *versus* ApoE4 carriers  on the production of ApoE fragments. The number of samples analyzed is indicated in parentheses.

Four different antibodies (ApoE 126-191, ApoE FL, ApoE Cter and ApoE 262–293) were used to characterize the ApoE fragments observed in cortex of AD patients (Fig. 4A). All fours antibodies recognized the 35 kDa form corresponding to the full length ApoE while none of the short ApoE fragments were recognized by the ApoE 126–191 antibody showing that all three fragments come from the COOH-half part of ApoE. The 16 and 18 kDa ApoE fragments lack the very C-terminus end of ApoE as shown by the lack of immunoreactivity with anti-ApoE Cter (Fig. 4A–C). The 12 kDa form is a C-terminal end form that is revealed by anti-ApoE FL, anti-ApoE 262–293 and anti-ApoE Cter antibodies (Fig. 4A–C).

We analyzed the fragments of ApoE found in ApoE2/3 carriers (including *APOE2/2, 2/3* and *3/3* genotypes) *versus* ApoE4 carriers (including *APOE3/4* and *APOE4/4* genotypes). The bar graph illustrated Fig. 4D show no difference between ApoE2/3 and ApoE4 carriers suggesting that the production of fragments of ApoE is not dependent on *APOE* genotype.

### Mixed ApoE-fragment/Aβ heteromers in AD brain

**Figure 5:**
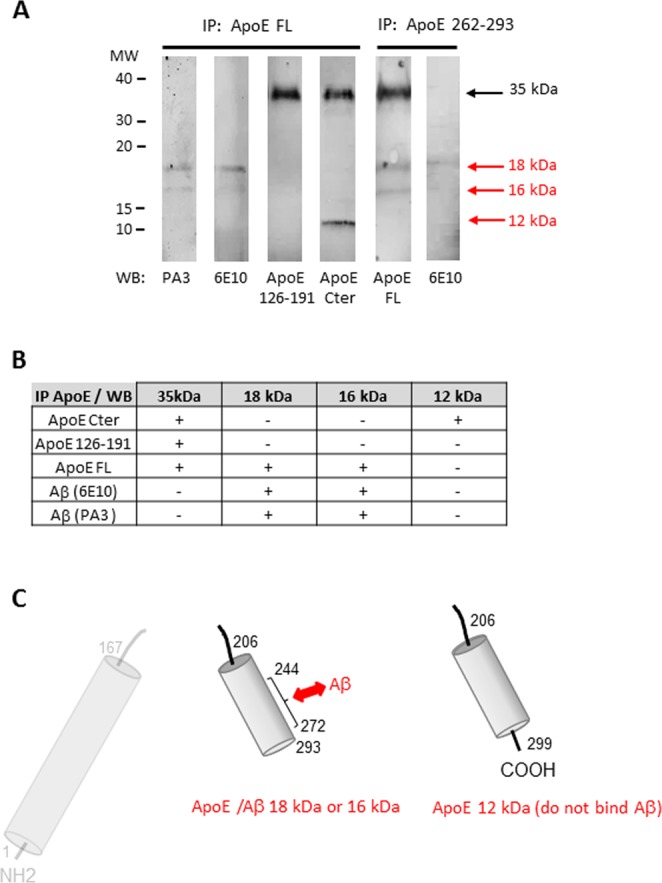
Co-immunoprecipitation of ApoE and Aβ revealed heteromers of 18 and 16 kDa composed of ApoE fragments  and Aβ in the cortex of AD patients. (**A**) Proteins extracted from human cortex of AD patients (Braak 6, Thal 4) were immunoprecipitated (IP) with an anti-ApoE FL or an anti-ApoE 262–293 antibody and western blotted (WB) with five anti-ApoE or anti-Aβ antibodies. The full length ApoE protein (35 kDa) was found in all samples revealed with anti-ApoE antibodies. In addition the 18, 16 and 12 kDa forms of ApoE were identified in the immunoprecipitates western blotted with appropriate anti-ApoE antibodies. The 18 and 16 kDa of these immunoprecipitates were also identified by PA3 and 6E10 antibodies as summarized in (**B**). The lanes illustrated in (**A**) have been cropped from western blots illustrated in Supplementary Fig. S3B. (**C**) Schematic illustration of ApoE regions involved in interaction with Aβ. In AD patients, ApoE fragments form heteromers with Aβ whose molecular weight are 18 and 16 kDa. In addition ApoE fragments of 12 kDa do not bind Aβ.

**Figure 6:**
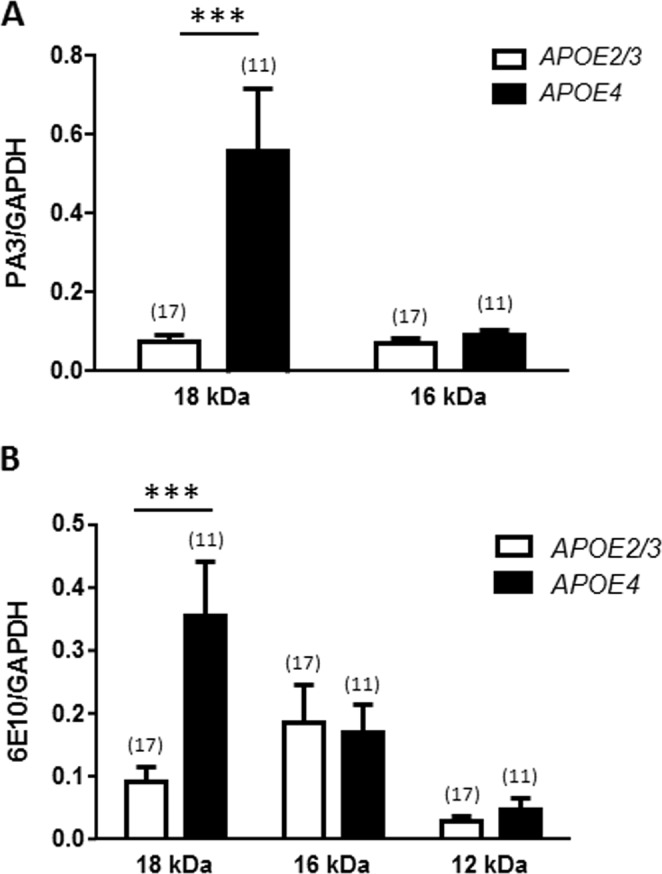
Effect of *APOE* genotype on the quantity of Aβ forms in the cortex of AD patients. Aβ 18 and 16 kDa measured with PA3 antibody (**A**) or with 6E10 antibody (**B**) in the cortex of patients carrying *APOE2/3* or *APOE4*. The number of samples analyzed is indicated in parentheses. Two way ANOVA and Bonferroni post-hoc analyses reveal a significant effect of *APOE4*-carriers on the production of the 18 kDa form of Aβ/ApoE heteromer with both Aβ antibodies (***p < 0.001).

Intrigued by the similarities of ApoE and Aβ profiles, we studied the interaction of ApoE fragments with Aβ in AD brains by co-immunoprecipitation (Fig. 5A). Anti-ApoE (FL and 262–293) were better capture antibodies than anti-Aβ antibodies to resolve Aβ forms bound to ApoE fragments. The full ApoE (35 kDa) and the 18, 16 and 12 kDa ApoE fragments were revealed by anti-ApoE Cter and anti-ApoE FL in the ApoE immunoprecipitates (Fig. 5A,B).The 18 and 16 kDa proteins precipitated with ApoE were also positive for Aβ western blotting (PA3 and 6E10) thereby revealing that they are composed of ApoE and Aβ. The 12 kDa form precipitated with ApoE was not recognized by any of the anti-Aβ antibodies (Fig. 6A,B). To sum up: in AD patients ApoE is fractioned and the ApoE fragments associate with Aβ to produce heteromers whose molecular weights are 18 and 16 kDa. In addition, small ApoE fragments of 12 kDa do not bind Aβ (Fig. 5C).

### More ApoE-fragment/Aβ heteromers in AD patient expressing APOE4 allele

Since *APOE4* allele is a high risk factor for developing AD, we analyzed the impact of *APOE* genotype on the quantity of Aβ in the cortex of patients (Fig. 6). ApoE4 carriers had significantly higher ApoE-fragment/Aβ 18 kDa levels measured with two different Aβ antibodies (PA3 in Fig. 6A, p < 0.0001 and 6E10 in Fig. 6B, p < 0.0001) while ApoE-fragment/Aβ 16 kDa was not different between *APOE* genotypes (Fig. 6A,B). These results show that the forms of ApoE-fragment/Aβ heteromers are not equally sensitive to the various *APOE* genotypes with the *APOE4* genotype producing more ApoE-fragment/Aβ 18 kDa. This result was unexpected when considering the fact that *APOE4* genotype did not increase ApoE 18 kDa fragments (Fig. 4D).

Since gender matters in AD (women with *APOE4* are more likely to develop AD than men with the same allele)^33^ and since our population of control patients was deficient in women and in *APOE4* allele carriers (Supplementary Table 1), we did similar analyze of ApoE fragment/Aβ heteromers restricted to the patients with AD (55.6% women and 55.6% *APOE4*). These analyzes confirm that *APOE4* genotype produces more ApoE-fragment/Aβ 18 kDa than *APOE2/3* (p = 0,0021 for analysis with PA3 and p = 0,0403 for analysis with 6E10; data not shown).

## Discussion

Physiological and pathological states of Aβ and ApoE in brain remain unclear, but here we show that 2 of the 3 forms of Aβ peptides specifically identified in the brain of AD patients are hybrid heteromers formed of Aβ1–42 peptides and ApoE middle fragments.

### 18, 16 and 12 kDa Aβ forms in the cortex of AD patients

Studies of post-mortem brain samples have revealed the increase of Aβ dimers and trimers^9,10^, dodecamers^9^ or tetra/pentamers^13,14^ in AD patients compared to control patients. Our results show specific increase of 16 and 18 kDa (which match tetra/pentamer’s size) and 12 kDa (which match trimer’s size) but no dodecamers in the brain of patients with AD. We did not detect Aβ monomer peptides (4 kDa) in their brain while we observed Aβ 4 kDa when purified Aβ42 peptides were used as external standard or when analyzing Aβ forms in the brain of APPxPS1 mice. Our results are consistent with other studies of human brain Aβ species^13,14,34^ and with study suggesting that Aβ appears as a tetramer that undergoes breakdown before plaque formation^35^. The 18 kDa Aβ form was also found in the hippocampus which confirms the importance of Aβ 18 kDa as a biomarker for AD. On the contrary, the absence of 16 kDa Aβ in the hippocampus is intriguing and requires larger hippocampal samples in control and AD patients to determine if the cortex and hippocampus have different Aβ signatures.

### Aβ1-42 peptides in AD patients

Antibodies targeting both the N-terminus and the C-terminus of Aβ1-42 identified the 18, 16 and 12 kDa Aβ forms indicating that all three Aβ forms in the brain of AD patients contain Aβ1-42. Previous trials aimed at identification of pathological Aβ forms have identified an assortment of Aβ species in oligomers^1,36^. Full Aβ1-42 peptides appear to be the earliest form to accumulate in the brain, and are thought to be modified over time into a complex array of truncated, isomerized, and/or phosphorylated peptides^37–39^. The diverse experimental procedures for protein preparations used in the different studies may modify the peptides and explain part of the diversity in Aβ species observed. Our results show that Aβ1-42 peptides are found in the three forms of Aβ observed in the brain of AD patient, which is consistent with previous work^4^, but we cannot exclude that other Aβ species may be part of the extracted peptides. Future experiments of immunoprecipitation combined with mass spectrometry should unmask more composites which could include several of the N- and C-terminally-truncated Aβ peptides identified in the brain of AD patients^4,7^.

### Different functions for the different Aβ forms

Correlative analyses between the quantities of each one of the Aβ forms with the MMSE of the patients showed that ApoE-fragment/Aβ 16 kDa heteromer and the Aβ oligomer 12 kDa correlate to the memory deficit while the ApoE-fragment/Aβ 18 kDa heteromer do not directly impact the memory. The difference between ApoE-fragment/Aβ heteromers 18 and 16 kDa probably retains important information relative to alteration of neuronal function. These forms may differ by the number and/or nature of Aβ they can bind^40^. Moreover, we could not exclude that ApoE-fragment/Aβ 18 kDa could play other pathogenic roles such as being intermediate Aβ storage forms or initiating tau hyperphosphorylation and neurodegeneration^28^.

We noticed that ApoE-fragment/Aβ 16 and Aβ 12 kDa correlate better with mid MMSE scores than with very low ones (MMSE < 10). This led us to speculate that in end-stage AD patients with lowest MMSE, neurodegeneration might play a major role in cognitive deficit. Whereas for early stages less demented patients (10 < MMSE < 24), the cognitive impairment might be due to Aβ-mediated alteration of neuronal function and might thus correlate with the quantity of Aβ forms.

### ApoE fragments in the brains of AD patients

Beside Aβ accumulation, ApoE fragmentation arose as a potential AD-related pathological process. We found three proteolytic fragments of ApoE in the brain of patient with AD: 18 and 16 kDa fragments coming from the COOH-half of ApoE lacking both the NH2-half and the C-terminus end and the 12 kDa fragment lacking the NH2-half but including the C-terminus end of ApoE. Previous studies have shown 12–20 kDa ApoE fragments in the brain of AD patients^29,41^ and a 19kDa C-terminal ApoE fragment that promote Aβ42 accumulation in human neuroblastoma and primary mouse neuronal cells^42^.

We did not detect N-terminal ApoE fragment in the brain of patient with AD suggesting that they are cleared, removed from the brain or degraded.

Our results show that the fragmentation of ApoE is independent of *APOE* genotype which disagrees with previous data showing that ApoE4 is more susceptible to proteolytic cleavage than ApoE3. These results were based on *in vitro* essays with recombinant ApoE3 or ApoE4 and *in vivo* in transgenic mice expressing ApoE3 or ApoE4^28,29^. In the studied population, which includes 75% heterozygous *APOE* genotypes, similar cleavage has been observed in *APOE4* and *APOE2/3* carriers. This is in agreement with statement that in addition to ApoE4, ApoE3 can also be cleaved by chymotrypsin-like serine protease which is elevated in AD^43^.

### ApoE-fragment/Aβ heteromers in human brain of patient with AD

Among the three forms of Aβ found in the brain of AD patients, the 18 and 16 kDa are ApoE-fragment/Aβ heteromers while the 12 kDa forms seem to be made of Aβ (probably trimers) which do not interact with the 12 kDa C-terminal ApoE fragments. The binding of ApoE fragments to Aβ could explain the higher molecular weight of ApoE 16/18 kDa compared to ApoE 12 kDa. Truncation of the C-terminus of ApoE (in ApoE-fragment/Aβ heteromers 16 and 18 kDa) seems to be an essential feature for the ability of the ApoE fragment to bind Aβ as it is for induction of AD-like neurodegeneration and behavioral deficits in transgenic mice^28^. After cleavage, ApoE fragments lacking the ApoE receptor-binding domain and the C-terminus, can still bind to Aβ and trap Aβ within the brain.

Molecular interactions between Aβ peptides and ApoE full length proteins have been described^17,19^. In brief, residues 12–28 of Aβ appear to contain the binding site for ApoE and residues 244–272 of ApoE are required for interaction with Aβ^23,44^. The site of Aβ-ApoE interaction is still present in fragments of ApoE produced in AD patients, but the N-terminal part of ApoE is lacking which invalidates the Aβ-clearance and favor Aβ accumulation.

Subtle differences in ApoE fragments or Aβ sequences composing the 18 and 16 kDa heteromers remain to be uncovered to explain their differences in molecular weight, in affinity for ApoE4 *versus* other ApoE and in ability to affect memory. The interaction between ApoE and Aβ seems to depend on ApoE isoform, its lipidation status, and the cellular compartment generating it^45^, suggesting that the role of the ApoE-fragment/Aβ heteromers could be determined by their intrinsic feature. The methods chosen for evaluating ApoE/Aβ complexes may greatly influence the results, for example ApoE purification, which delipidates ApoE, modifies the ApoE to Aβ binding^46^. It is likely that any protein preparation that either purify or denature proteins will end up with low or no detection of ApoE-fragment/Aβ heteromers.

### Role of APOE4 in ApoE-fragment/Aβ interaction

Our data show that the *APOE4* genotype has a great propensity to produce 18 kDa ApoE-fragment/Aβ forms and suggest that the fragments derived from ApoE4 carriers could better associate with Aβ to form ApoE/Aβ 18 kDa heteromers. A simple explanation could have been that ApoE4 carrier’s produce more ApoE fragments^28,29^, but this hypothesis is ruled out by our results showing no significant difference of ApoE fragment production between ApoE4 and ApoE2/3 carriers. Another bias could be the low representation of women and ApoE4 carriers in the control group, however, analyzes performed on a population restricted to AD patients and composed of half women and half carrier ApoE4, also argue that ApoE4 increases the ApoE fragment/Aβ 18 kDA heteromers.

Low lipidation of ApoE4 has been suggested to result in reduced ApoE4/Aβ levels and increased accumulation of Aβ^47^. Our results suggest that, if lipidation of ApoE4 fragments is altered, it is rather favoring its binding to Aβ resulting in increased formation of ApoE-fragment/Aβ heteromers and increased AD risk. Understanding the interplay between ApoE4 and Aβ seems important because neither ApoE4 nor Aβ on alone does drive risk of memory loss in human^48^.

## Conclusions

Our results show that ApoE fragments lacking N- and C-termini are partners of Aβ in inducing AD pathology. Because they lack the Aβ transporter-binding domain, ApoE-fragment/Aβ heteromer formation slow down Aβ clearance and promote Aβ accumulation within the brain of patients with AD. *APOE4* is more efficient than other *APOE* genotype for ApoE-fragment/Aβ heteromer formation. Although N-terminal fragments of ApoE is not correlated with AD in the plasma or CSF^49^, other ApoE fragments and ApoE-fragment/Aβ heteromers might be targeted in brain and peripheral fluids of AD patients to define strategies to detect or reverse AD. A promising strategy to decrease AD progression could be to control ApoE fragmentation which should in turn revive the clearance of Aβ and decrease deleterious effects of Aβ and ApoE fragments in the brain.

## Methods

### Brain collection

Post-mortem human cortical brain tissues were obtained from Neuro-CEB brain bank of Hôpital de la Pitié-Salpétrière (Paris, France) after informed consent of all participants during their life and/or their legal guardians, human hippocampal brain tissues were obtained from the Bio-bank of the anatomo-pathology department of CHU (Bordeaux, France) after ethics approval and consent. All the experiments were done according to the legislation in force for the use of biological samples in scientific research and respecting the anonymity of the donors.

Since definitions of AD are based on the co-occurrence of Aβ and tau pathologies, post-mortem brain tissues have been classified after neurofibrillar tau and Aβ plaque scoring by members of the Neuro-CEB network as follow: Braak stage 4/6 and Thal stage 2/5 for AD patients and Braak stage 0/2 and Thal stage 0/1 for control patients. Four samples displaying other pathologies (Parkinson disease, Fronto-temporal dementia) and ambiguous staging (high Braak and low Thal or High Thal and low Braak) were discarded from the study. Characteristics of the population of patients whom brain were analyzed in this study were listed in Supplementary table 1. Ten of these cases had been clinically diagnosed according to MMSE score (Mini-Mental State Examination). The MMSE and psychiatric history of others patients are not known. All brain samples were collected at Neuro-CEB or CHU Bordeaux and immediately stored at −80 °C until use.

We also used brain samples from 5 APPxPS1 male mice aged 8–9 months old. These mice overexpress the human PS1dE9 mutant plus the human APP displaying the double Swedish mutation and show memory deficits and Aβ plaques starting around 4–5 months old^50^. The brain were collected after cervical dislocation of the mouse and stored at −80 °C immediately after collection and until use.

### Biochemical analyses of brain tissues

We designed protein extraction and Western blot conditions to preserve the native structure of the Aβ (no SDS, no formic acid, keep 4 °C temperature throughout the duration of protein preparation). In these conditions, the β-sheet folded Aβ plaques stained by Thioflavine S could not migrate into the gel and were found in deposit pits only. Similar post-mortem intervals between death and brain sampling were respected in AD and control patients (see Supplementary Table 1). Therefore post-mortem interval was not identified as a confounding factor and we did not exclude any sample on this basis. ApoE fragment/Aβ heteromers were observed in all AD patients but they were never observed in control patients even those with the longer post-mortem intervals. These observations exclude the possibility that ApoE fragment/Aβ heteromers could be generated during post-mortem protein degradation. For some control experiments (see Supplementary Fig. S1) the protein extraction were performed in the presence of 2% SDS, 10% formic acid or the native proteins were leave overnight at room temperature for gentle degradation.

Frozen brain tissues were homogenized in two volumes of ice cold lysis buffer (HEPES 20 mM pH 7.2; NaCl 100 mM; Triton 0.5%, Protease Inhibitor Cocktail 1%) and shaken with silicon beads (diameter 1,4 mm) in Minilys (Bertin; 5000 rpm, 4 cycles of 15 s separated by ice cooling). After testing centrifugations of the samples, we decided to keep the whole homogenates to preserve all forms of Aβ and store the preparations at −80 °C. The protein concentration was determined by DC protein assay kit (Bio-Rad) and by direct 280 nm UV measurement (Biodrop, Fisher Scientific), it was adjusted to 10 µg/µL and stored at −20 °C before electrophoresis.

Brain homogenates (50 µg of total protein) or purified Aβ42 peptides (ref 1428, Tocris, 0.1 µg) were electrophoretically resolved in a precast 4–20% Tris-Glycine Criterion gels (TGX, BioRad) for 45 min at 180 V. Proteins were transferred from gel onto PVDF membranes (Transblot, BioRad). Before 6E10 immunostaining, membranes were left during 15 min in PBS at 85 °C for epitope unsmasking and wash in PBS-Tween. For all immunostaining, membranes were incubated for 1 h at room temperature in 5% non-fat dry milk to block unspecific binding.

Immunodetection of the proteins was performed by incubating the membranes for 24 h at 4 °C with primary antibodies against Aβ: 6E10 (AB_662799, Covance), PA3 (AB_2258328, Thermo Scientific), G2–11 (AB_10562244, Millipore), MOAB2 (AB_2313888, Millipore) or antibodies against ApoE: ApoE FL (AB_564230, Calbiochem,), ApoE 126–191 and ApoE C-ter (sc-393302, SantaCruz), ApoE 262–293 (AB_2057990, Abgent). Secondary antibodies, dilutions and conditions used for IR imaging are summarized in Supplementary Table 2.

Western blots were imaged with the Odyssey scanner (LiCor) and analyzed with Image Studio Software (LiCor). Anti-GAPDH antibody (AB_10615768, Millipore) was used as loading control and allowed the quantification of Aβ and ApoE as x/GAPDH ratios.

### Co-immunoprecipitation

For co-immunoprecipitation of ApoE and Aβ, we followed the Pierce classic Magnetic IP Kit instructions. Briefly, equal amounts of total homogenates (1500 μg) were incubated with 10 µg of IP antibody (anti-ApoE FL antibody, anti-ApoE 262–293, PA3 or 6E10 antibodies) for 1–2 h at room temperature (RT) or overnight at 4 °C. Antigen/antibody complex was bound to Protein A/G magnetic beads for 1 h at RT. Non-bound sample components were washed away, and antibody and antigen were eluted with a buffer that disrupts the binding interactions. The antigens were further characterized by classical ApoE or Aβ Western blot as described above.

### Apolipoprotein-E genotyping

We have used restriction enzyme isoform genotyping for rapid typing of *APOE (E2, E3, E4)* alleles in the human brain samples used in this study. *APOE* restriction isotyping used oligonucleotides to amplify *APOE* gene sequences encoding for amino acid positions 112 and 158. The amplification products were digested with *Cfo1* and subjected to electrophoresis on polyacrylamide gels. Each of the isoforms was distinguished by a unique combination of *Cfo1* fragment sizes as previously reported^51^.

### Statistical analyses

GraphPad Prism 6.0 was used for all statistical analysis. The peptides observed in the brain of control patients (Braak 0/2 and Thal 0/1 which express few if any tangles and plaques) were compared to those of AD patients (Braak 4/6 and Thal 2/5 which display significant amount of tangles and plaques). Data were analyzed with the Mann-Whitney test for comparison of the same peptide between control and AD and with two-way ANOVA for comparison of different forms of peptides between *APOE2/3* versus *APOE4* genotypes. Correlation analysis was conducted using Pearson’s correlation. All Data were presented as the mean ± SEM (Standard Error of the Mean) or in plots where each data point represent one subject, p values < 0.05 were considered to be significant.

### Ethics approval

Human brain tissues were obtained from Neuro-CEB brain bank of Hôpital de la Pitié-Salpétrière, Paris, France and from the Bio-bank of the anatomo-pathology department of CHU Bordeaux, France after ethics approval and consent of the Committee for the Protection of Persons (CPP No. CEBH 2009/03; MESR: DC-2008-337).

## Supplementary information

Supplementary information

## Data Availability

The datasets used and/or analyzed during the current study are available from the corresponding author on reasonable request.
