# Supplementary material for: ApoE-fragment/Aβ heteromers in the brain of patients with Alzheimer’s disease
Source: Sci Rep. 2019 Mar 8;9:3989. doi: 10.1038/s41598-019-40438-4 (PMC6408522; doi:10.1038/s41598-019-40438-4)
Supplement: Supplementary file 1 — Supplementary information [file 41598_2019_40438_MOESM1_ESM.pdf]

**ApoE-fragment/Aβ heteromers in the brain of patients with Alzheimer's disease**

Amandine Mouchard, Marie-Charlotte Boutonnet, Claire Mazzocco, Nathalie Biendon, Neuro-CEB Neuropathology Network, Nathalie Macrez.

## Supplementary informations include:

Table 1: Characteristics of the patients whose Cortex was studied.

Table 2: Antibodies used in the study

Figure S1: Control experiments for identification of Aβ forms.

Figure S2: Aβ 18 kDa is also found in hippocampus of AD patients.

Figure S3: Full Western blots.

Supplementary Table 1: Characteristics of the patients whose Cortex was studied

| | **controls** | **AD** |
|---|---|---|
| n | 12 | 18 |
| Gender, % women | 16.7 | 55.6 |
| Age, years old (± sem) | 75.2 (± 3.3) | 73.2 (± 2.6) |
| MMSE (± sem) | 25[d] | 11.1 (± 2.4)[e] |
| Braak stage[a] (± sem) | 1.1 (± 0.3) | 5.7 (± 0.1) |
| Thal stage[b] (± sem) | 0.3 (± 0.2) | 3.8 (± 0.3) |
| *APOE4*[c], % patients | 8.3 | 55.6 |
| PMI (± sem) | 28 (± 5.0) | 25 (± 2.6) |

AD: Alzheimer's disease

PMI: Post-mortem interval (hour)

[a] ranked between 0 and 6 according to Braak & Braak, 1991

[b] ranked between 0 and 5 according to Thal et al., 2002

[c] % of patients having at least one *APOE4* allele

[d] only one value for a patient aged 86 years old, the other control patients have not been tested for MMSE

[e] nine patients out of 18 were tested for MMSE

Supplementary Table 2: Antibodies used in the study

| primary Ab I | immunogen | ref | dilution I | secondary Ab II | ref | dilution II |
|---|---|---|---|---|---|---|
| PA3 | full length Aβ1-42 | AB_2258328 | 1/2000 | goat anti-rabbit IRDye 800 | AB_621843 | 1/5000 |
| 6E10 | hu Aβ3-8 | AB_662799 | 1/2000 | goat anti-mouse Alexa Fluor®680 | AB_2535723 | 1/2000 |
| G2-11 | hu Aβ35-42 | AB_10562244 | 1/1000 | goat anti-mouse Alexa Fluor®680 | AB_2535723 | 1/2000 |
| MOAB2 | full length Aβ1-42 | AB_2313888 | 1/1000 | goat anti-mouse Alexa Fluor®680 | AB_2535723 | 1/2000 |
| APOE FL | recombinant hu APOE | AB_564230 | 1/1000 | donkey anti-goat Alexa Fluor®680 | AB_2535741 | 1/2000 |
| ApoE 126-191 | hu ApoE126-191 | AB_626691 | 1/1000 | goat anti-mouse Alexa Fluor®680 | AB_2535723 | 1/2000 |
| ApoE 262-293 | hu ApoE262-293 | AB_634032 | 1/1000 | goat anti-rabbit IRDye 800 | AB_621843 | 1/5000 |
| ApoE C-ter | hu ApoE 293-318 | Sc-393302 | 1/1000 | goat anti-mouse Alexa Fluor®680 | AB_2535723 | 1/2000 |
| GAPDH | hu GAPDH | AB_10615768 | 1/5000 | donkey anti-chicken IRDye680 or 800 | AB_10707008 or AB_1850023 | 1/5000 |

AB_ref, Antibody Registory reference ; hu, human

**Supplementary Figure S1:**

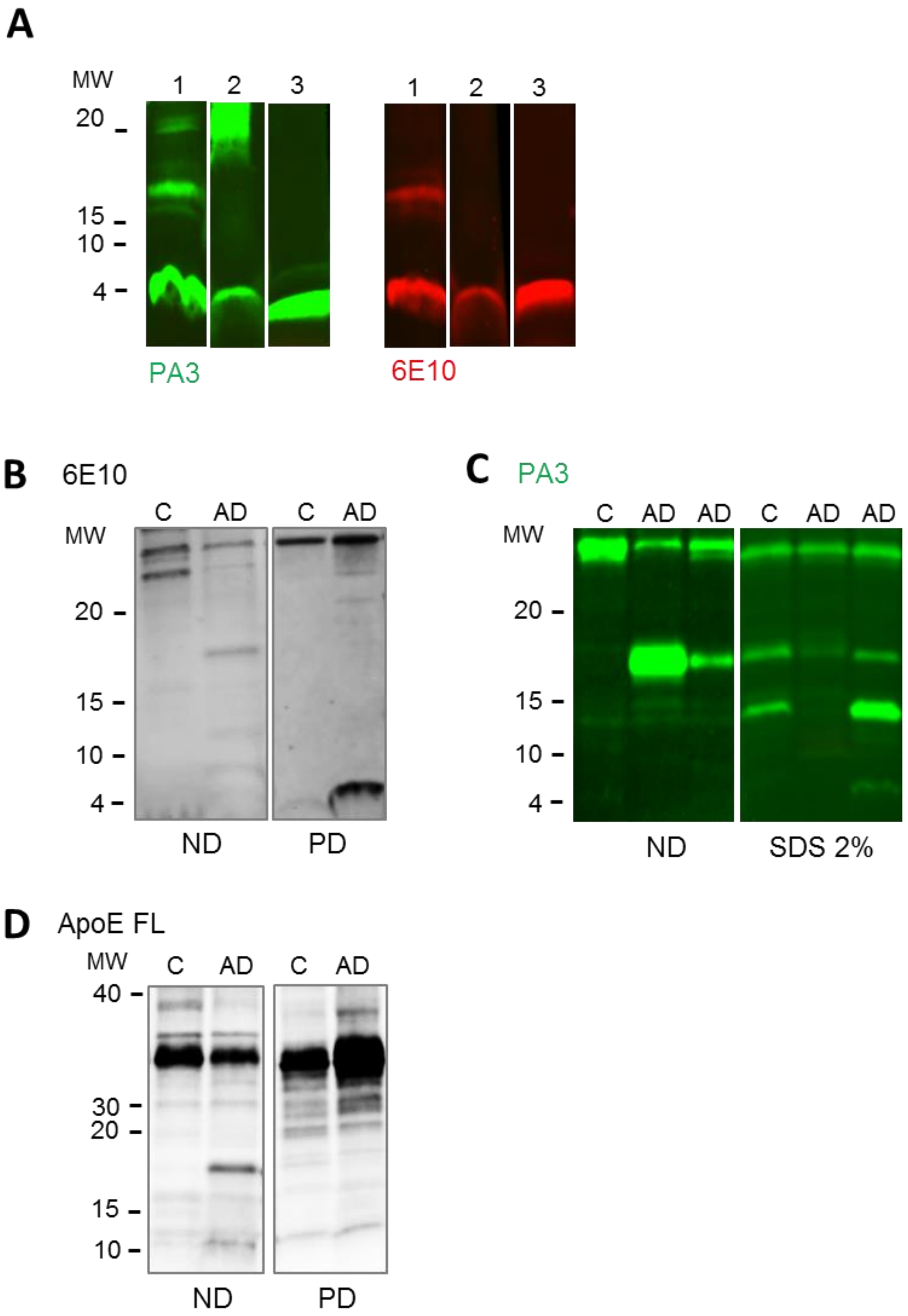


**Figure S1: Control experiments for identification of Aβ forms. A**, Western blot of synthetic human Aβ42 (lane 3), non-denaturant brain extract from APPxPS1 mouse (lane 1) and brain extract from APPxPS1 mouse prepared in the presence of formic acid 10% (lane 2) revealed by PA3 or 6E10 antibodies. Western blot of proteins extracted from control (C) versus AD human cortex and revealed with 6E10 (**B**) or PA3 (**C**), after various protein preparations in non-denaturant conditions (ND), in

denaturant conditions (SDS 2%) or after protein degradation (PD; overnight at room temperature). **D**, Western blot of proteins extracted from human cortex (C, control *versus* AD) and revealed with ApoE FL, after protein preparations in non-denaturant conditions (ND) or after protein degradation (PD; overnight at room temperature).

## Supplementary Figure S2:

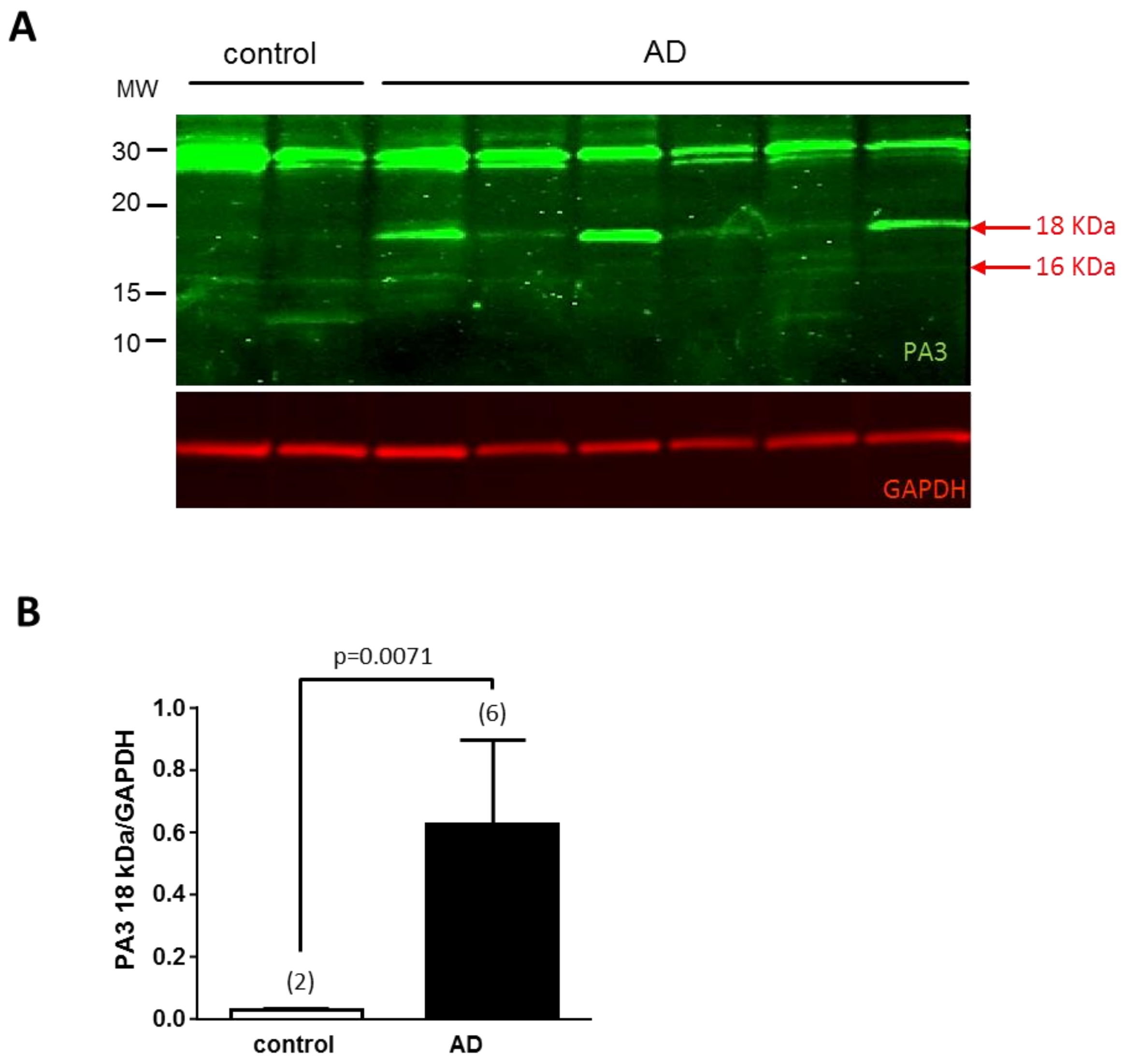


**Figure S2: Aβ 18 kDa is also found in hippocampus of AD patients. A**, Western blot of proteins extracted from hippocampus of control and AD patients and revealed with the full length anti-Aβ (PA3-16761 in green). GAPDH (in red) was revealed as an internal control for each deposit of the western blot. **B**, Statistical analysis of Aβ 18kDa/GAPDH ratio measured in control *versus* AD brain samples. Despite a low statistical probability that this effect may be random ($p = 0.0071$), the huge effect is not considered significant when analyzed with the Mann Whitney test because of the small number of control samples ($n = 2$).

## Supplementary Figure S3:

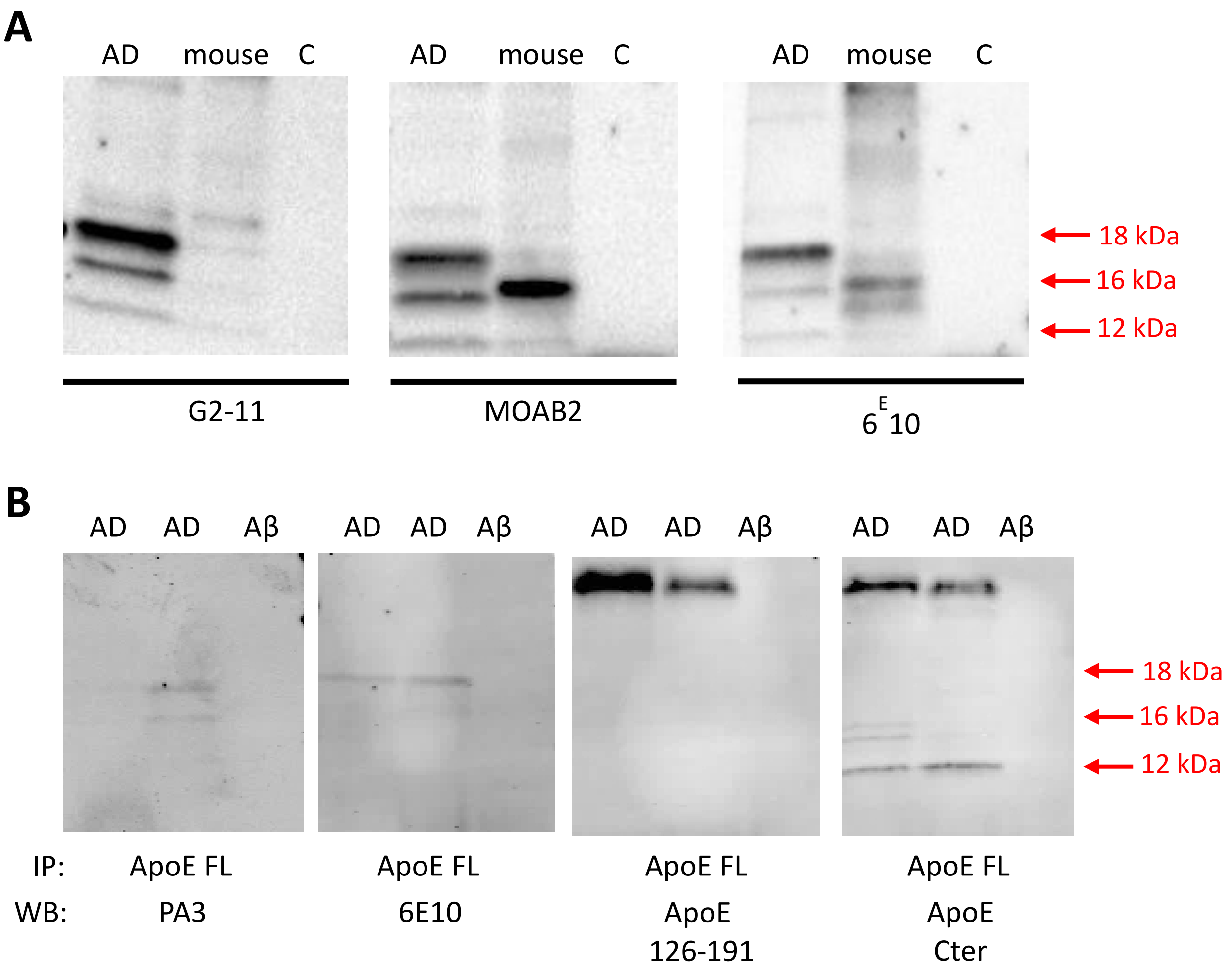


**Figure S3: Full western blots. A**, Western blots of AD human cortex (AD), brain extract from APPxPS1 mouse (mouse) and control human cortex (C) revealed with G2-11, MOAB2 and 6E10. **B**, Western blots of AD human cortex (AD) or synthetic Aβ (Aβ) after ApoE FL immunoprecipitation immunoblotted with PA3, 6E10, ApoE 126-191, ApoE Cter as indicated.
